# Correction: Investigating the association between diabetes and carpal tunnel syndrome: A systematic review and meta-analysis approach

**DOI:** 10.1371/journal.pone.0326976

**Published:** 2025-06-24

**Authors:** Elaheh Sanjari, Hadi Raeisi Shahraki, Lusine G. Khachatryan, Abdollah Mohammadian-Hafshejani

The initials of the third author are indexed incorrectly in PubMed. The correct initials are: Khachatryan LG.

The correct citation is: Citation: Sanjari E, Raeisi Shahraki H, Khachatryan LG, Mohammadian-Hafshejani A (2024) Investigating the association between diabetes and carpal tunnel syndrome: A systematic review and meta-analysis approach. PLoS ONE 19(4): e0299442. https://doi.org/10.1371/journal.pone.0299442
